# Characterization, evolutionary analysis, and expression profiling of the* VrPYL* gene family in mung bean in response to abiotic stress

**DOI:** 10.7717/peerj.21432

**Published:** 2026-06-22

**Authors:** Lili Yin, Yonglin Xiao, Jiaqi Liao, Dongxu Zhang, Xiaoliang Chen

**Affiliations:** 1 College of Agronomy and Life Sciences, Shanxi Datong University, Datong, Shanxi, China; 2 Facility Agriculture Technology Research and Development Center, Shanxi Datong University, Datong, Shanxi, China; 3 School of Medicine, Shanxi Datong University, Datong, Shanxi, China; 4 Institute of Respiratory and Occupational Diseases, Shanxi Datong University, Datong, Shanxi, China

**Keywords:** Mung bean, *PYL* gene family, Abscisic acid, Abiotic stress

## Abstract

Abscisic acid (ABA) plays a central role in regulating plant growth and development and in mediating responses to both biotic and abiotic stresses. PYL proteins serve as the core receptors in the ABA signaling pathway. The characteristics of the *PYL* gene family and its response to abiotic stresses in mung bean (*Vigna radiata* L.) have not been clarified. In this study, we performed genome-wide screening of mung bean and identified nine *PYL* genes unevenly distributed across six chromosomes. Bioinformatic predictions revealed that all encoded VrPYL proteins are predicted to be hydrophilic and primarily localized to chloroplasts and the cytoplasm. Segmental duplication was the primary driver of the expansion of this gene family, with duplicated gene pairs having undergone purifying selection during evolution. Phylogenetic analysis showed that VrPYL proteins were divided into three groups, and members within each group exhibited conserved exon-intron structures and motif composition. Homology and synteny analyses indicated that the *PYL* gene family has remained relatively conserved among mung bean and other plant species. A protein domain analysis demonstrated that all VrPYL proteins contained the “helix-clamp” domain typical of the* PYL* gene family. Notably, the third and fourth amino acid residues within the CL2 region exhibited marked polymorphism: the valine-valine (VV) motif was the predominant combination, while the valine-threonine (VT) variant displayed mung bean-specific characteristics. Cis-element analysis of the *VrPYL* gene promoters revealed diverse hormone-responsive and stress-responsive elements. Furthermore, qRT-PCR assays showed that most *VrPYL* genes responded to ABA, PEG and salt stress treatments, with *VrPYL3* and *VrPYL4* emerging as promising candidate genes in response to drought and salt stress. Overall, this study lays a foundation for investigating the functions of *VrPYL* genes in abiotic stress responses and provides valuable genetic resources for molecular breeding.

## Introduction

Abscisic acid (ABA) is one of the key hormones regulating plant growth and development. Studies have shown that ABA is involved in cell elongation and division, seed dormancy, plant senescence, leaf abscission, and other physiological processes (Bartels & Sunkar, 2005; Fujii, Verslues & Zhu, 2007). Additionally, ABA mediates plant adaptive reactions to both biotic and abiotic stressors (Lee & Luan, 2012; Zhu, 2002). The ABA signal transduction pathway is primarily composed of three core components: the ABA receptor PYR/PYL/RCAR family, type 2C protein phosphatases (PP2Cs) serving as negative regulatory factors, and sucrose non-fermenting 1-related protein kinase 2 (SnRK2) acting as positive regulatory factors (Kobayashi et al., 2005; Ma et al., 2009; Park et al., 2009). Under normal physiological conditions, PP2Cs specifically binds to SnRK2s, catalyzing the dephosphorylation of serine/threonine residues on SnRK2s. This results in the loss of protein kinase activity of SnRK2s, and the ABA signaling pathway is subsequently silenced (Vlad et al., 2009). When plants are exposed to external stimuli, the endogenous ABA content increases and binds to PYL proteins (Johnson et al., 2002). This binding induces a conformational change in PYL proteins, enabling them to interact with the downstream negative regulatory factors PP2Cs (Furihata et al., 2006; Melcher et al., 2009). The interaction between PYL proteins and PP2Cs relieves the inhibitory effect of PP2Cs on SnRK2s, thereby activating SnRK2s (Lumba, Cutler & McCourt, 2010; Santiago et al., 2009). Then, SnRK2s phosphorylate downstream target proteins, which in turn trigger the ABA signaling pathway (Fujii, Verslues & Zhu, 2007).

Terrestrial plants have evolved ABA-activated receptors to cope with abiotic stresses (Fujii et al., 2009). The PYL family constitutes the largest characterized family of plant hormone receptors (Dupeux et al., 2011). In *Arabidopsis thaliana*, ABA is perceived by the soluble cytosolic Pyrabactin Resistance 1 (PYR1)/Pyrabactin Resistance 1-Like (PYL)/ABA Receptor Component (RCAR) protein family, which comprises 14 functional genes (Bai et al., 2013). Homologous genes of the PYR/PYL/RCAR family have been identified and characterized in various crop species, including 13 PYL members in rice, 21 in soybean, 27 in cotton, 14 in tomato, and 8 in grape (Boneh et al., 2012; Chen et al., 2017; González-Guzmán et al., 2014; Yadav et al., 2020; Zhang et al., 2020). Functional characterization of multiple *PYL* genes has further corroborated their roles in plant biology. Specifically, overexpression of *AtPYR1*, *AtPYL1*, *AtPYL2*, and *AtPYL3* in *A. thaliana* confers enhanced drought tolerance and improved water use efficiency (Li et al., 2018). Furthermore, *AtPYL5* and *AtPYL9* have been demonstrated to mediate both drought and cold tolerance (Lenka et al., 2018). In rice, overexpression of *OsPYL5* and *OsPYL10* augments drought and cold tolerance (Kim et al., 2014). In maize, overexpression of *ZmPYL8*, *9*, and *12* enhances cold stress tolerance (He et al., 2018). In wheat, overexpression of *TaPYL* genes increases ABA responsiveness, reduces transpiration, and increases the photosynthetic rate, thereby markedly reducing water requirements (Mega et al., 2019). Taken together, these lines of evidence underscore that *PYL* genes serve as key regulators in mediating plant resistance to abiotic stresses.

Mung bean (*Vigna radiata* L.), an annual herbaceous plant belonging to the genus *Vigna* of the *Fabaceae* family, is a globally cultivated legume crop with both nutritional and medicinal value. Mung bean is grown on arid and barren soils, serving as an excellent stress-tolerant crop that thrives in adverse environments (Kang et al., 2014). However, drought and salinization have reduced mung bean yield and quality. Therefore, there is an urgent need to develop drought- and salt-tolerant mung bean cultivars, and achieving this goal requires screening functional genes that endow plants with salt and drought resistance. The ABA signaling pathway is crucial for plants to withstand abiotic stress, and among its key components are PYL receptors (Vishwakarma et al., 2017). The *PYL* gene family in mung bean has not yet been characterized. Thus, in this study, we carried out a comprehensive identification and analysis on the mung bean genome, with nine *VrPYL* genes successfully characterized. Additionally, we analyzed the phylogenetic relationships between these *VrPYL* genes and *PYL* genes from other species, and characterized the physicochemical properties, gene duplication and expansion patterns, gene structures, motif composition, and cis-acting elements of the *VrPYL* gene family. Furthermore, *PYL* gene synteny across species was investigated, and *VrPYL* expression patterns under stresses were analyzed *via* qRT-PCR. The results of this study have deepened our understanding of the functions of *VrPYL* genes under various stress conditions and provide a foundation for screening target genes that can improve the abiotic stress tolerance of mung bean.

## Materials & Methods

### Genome-wide identification of the *VrPYL* gene family

Genome-wide protein sequences of mung bean were downloaded from the Ensembl Plants database (http://plants.ensembl.org/index.html). Using the PYL protein sequences of *Arabidopsis thaliana* (AtPYL) as references, we performed BLASTP to search against the mung bean protein database with an *E*-value threshold of 1e^−5^ (Altschul et al., 1990), yielding candidate PYL proteins of mung bean. Subsequently, the Hidden Markov Model (HMM) file of the polyketide_cyc2 domain (accession number: PF10604) was downloaded, and HMMER 3.0 software (with default parameter settings) was used to screen PYL proteins from the aforementioned mung bean protein sequences (Finn, Clements & Eddy, 2011). Candidate members obtained from the two methods were merged, and redundant sequences were removed. The merged non-redundant set included a small number of candidates identified by only one of the two methods. The resulting sequences were submitted to the SMART database (http://smart.embl-heidelberg.de/) (Letunic, Doerks & Bork, 2015), NCBI Conserved Domain Database (CDD, https://www.ncbi.nlm.nih.gov/cdd), and Pfam database (http://pfam.xfam.org/) (Wilkins et al., 1999) to verify the presence of PYR/PYL (RCAR)-like domains. Only sequences containing complete PYR/PYL (RCAR)-like conserved domains were retained as the final *VrPYL* genes (nine in total). The *VrPYL* genes were named according to their sequential chromosomal locations. Gene IDs and chromosomal location data of *VrPYL* genes were retrieved from the mung bean genome annotation database, and MapChart software was used to generate the chromosomal distribution map of *VrPYL* genes.

### Sequence analysis and basic information of the *VrPYL* gene family

The molecular weight (MW), theoretical isoelectric point (pI), amino acid (aa) length and other physical and chemical information of *VrPYL* genes were calculated on the ExPASy server (http://web.expasy.org/). Subcellular localization was predicted using the PLoc server (http://www.csbio.sjtu.edu.cn/bioinf/Cell-PLoc-2/).

### Phylogenetic analysis of the *VrPYL* gene family members

Phylogenetic trees were generated with MEGA 7.0 software based on PYL protein sequences derived from mung bean, *A. thaliana*, rice, soybean, *Brachypodium distachyon* and apple (Data S1). First, multiple sequence alignment of PYL protein sequences from the aforementioned species was performed using ClustalW software (Thompson, Higgins & Gibson, 1994). Subsequently, phylogenetic tree was built based on the neighbor-joining method, and maximum likelihood with 1,000 bootstrap replicates was used for validation (Kumar, Stecher & Tamura, 2016).

### Collinearity analysis of the *PYL* gene family

Genome sequences and their corresponding annotation files for mung bean, *A. thaliana*, rice and soybean were downloaded and used to investigate the genetic relationships among *PYL* genes across different species. Multiple sequence alignment was used to analyze the similarity of *PYL* genes between the mung bean and three other species. MCScanX software was employed to identify syntenic blocks among different species, generating a synteny output file (Wang et al., 2012). The analyzed gene pairs were mapped to their chromosomal positions to produce visualization files of gene-pair synteny, which were subsequently used for plotting.

Synteny analysis of *VrPYL* genes was performed using McScanX, and Circos was employed to construct the synteny relationship map (Krzywinski et al., 2009). Calculator 2.0 was used to obtain the non-synonymous substitution rate (Ka) and synonymous substitution rate (Ks) of duplicated gene pairs in the *VrPYL* gene family, and the Ka/Ks ratio was applied to estimate the selection pressure acting on the duplicated genes (Wang et al., 2010).

### Exon-Intron structure, conserved motifs, and protein feature analyses of *VrPYL* genes

The coding sequences and full-length genomic sequences of nine *VrPYL* genes were uploaded to the Gene Structure Display Server (https://gsds.cbi.pku.edu.cn/) for exon-intron structural analysis (Guo et al., 2007). Conserved motifs within these nine VrPYL proteins were analyzed using the MEME web server (http://meme-suite.org/tools/meme) (Bailey et al., 2009).

Multiple sequence alignment of VrPYL proteins was conducted using ClustalW software. The crystal structure of AtPYR1 (PDB ID: 3K90) was obtained from the RCSB Protein Data Bank (https://www.rcsb.org/). Using this structure as a template and integrating the alignment results, we predicted the secondary structures of VrPYL proteins using the ESPript server (https://espript.ibcp.fr/ESPript/cgi-bin/ESPript.cgi).

### Cis-acting element analysis of *VrPYL* gene promoters

For the prediction of cis-acting elements within *VrPYL* gene promoters, 1,500-bp sequences upstream of the translation initiation codon (ATG) of each *VrPYL* gene were initially extracted from the mung bean genome and defined as the promoter sequences of the target genes. Subsequently, the PlantCARE web server (http://bioinformatics.psb.ugent.be/webtools/plantcare/html/) was used to analyze the cis-acting elements in the promoter sequences (Lescot et al., 2002).

### Plant materials and treatments

Plump mung bean seeds (cultivar: VC1973A) were pre-germinated and subsequently transferred to 1/2 MS medium for hydroponic cultivation. The growth conditions were maintained at 24 °C with a 16-h light and 8-h dark photoperiod. Following full expansion of the first trifoliolate leaf, the seedlings were exposed to hormone and abiotic stress treatments by adding the corresponding treatment reagents directly into the hydroponic nutrient solution. Three experimental groups were implemented: 100 µmol/L ABA, 15% PEG-6000, and 100 mmol/L NaCl. Leaf samples were collected at 0, 6, 12, and 48 h following treatment. Fresh samples were flash-frozen in liquid nitrogen immediately and stored at −80 °C after harvesting for subsequent total RNA isolation and gene expression analysis. Each sample consisted of leaves from three independent plants and three experiments were performed.

### RNA isolation and quantitative reverse transcription PCR

Total RNA was isolated from frozen leaf samples using an RNAprep Pure Plant Kit (Tiangen Biochemical Technology, Beijing, China). The integrity of extracted RNA was confirmed by agarose gel electrophoresis, which yielded clear 28S and 18S rRNA bands under UV light. Meanwhile, RNA concentration and purity were determined using a NanoDrop ONE spectrophotometer (Thermo Fisher Scientific, Waltham, MA, USA), and samples with concentration ≥ 150 ng/µL, 260/280 nm absorbance ratios within the range of 1.8 to 2.0, and 260/230 nm absorbance ratios ≥ 2.0 were considered acceptable. First-strand complementary DNA (cDNA) was synthesized from total RNA using the Takara PrimeScript™ RT reagent (with gDNA Eraser) to eliminate genomic DNA contamination. Gene-specific quantitative primers for *VrPYL* genes were designed *via* Primer3Plus (https://primer3plus.com/) and synthesized commercially; the primer sequences are listed in Data S2. SYBR Green-based qPCR was performed using a SYBR Green qPCR Kit (Tiangen Biochemical Technology, Beijing, China) with the following thermal cycling program: an initial denaturation at 95 °C for three min, followed by 40 cycles of 95 °C for 10 s and 60 °C for 40 s. The *VrACTIN3* gene (*Vradi03g00210*) was employed as the internal control gene, with relative expression levels of *VrPYL* genes quantified by the 2^−ΔΔCt^ method (Livak & Schmittgen, 2001). Each biological sample was analyzed with at least three technical replicates.

## Results

### Genome-wide identification and chromosomal localization of the *VrPYL* gene family

In this study, nine *PYL* genes were characterized in mung bean and named *VrPYL1*–*VrPYL9* according to their physical positions on the chromosomes. Analysis of their physicochemical properties (Table 1) revealed that the VrPYL proteins varied in amino acid composition. These proteins had sequence lengths spanning 148 (VrPYL9) to 280 (VrPYL2) aa, along with MW of 16.29–31.58 kDa and theoretical pI of 4.72–9.35. All VrPYL proteins were acidic except for VrPYL1/2/3, which were alkaline. The grand average of hydropathicity of all VrPYL members was less than 0, indicating that they were all hydrophilic proteins. Most VrPYL proteins were predicted to localize to the chloroplast and cytoplasm, with only VrPYL4 predicted to be localized in the nucleus. Furthermore, the distribution of *VrPYL* genes across the 11 mung bean chromosomes was analyzed using annotation files from the mung bean genome database. The nine *VrPYL* genes were distributed across chromosomes 2, 5, 6, 7, 9, and 10 (Data S3), with five chromosomes lacking *PYL* genes. Chromosome 6 harbored the most *PYL* genes (VrPYL3/4/5/6), while the other chromosomes contained only one *PYL* gene each. Additionally, most *VrPYL* genes were distributed in the terminal regions of the chromosomes.

### Phylogenetic analysis of the *VrPYL* gene family

**Figure 1:**
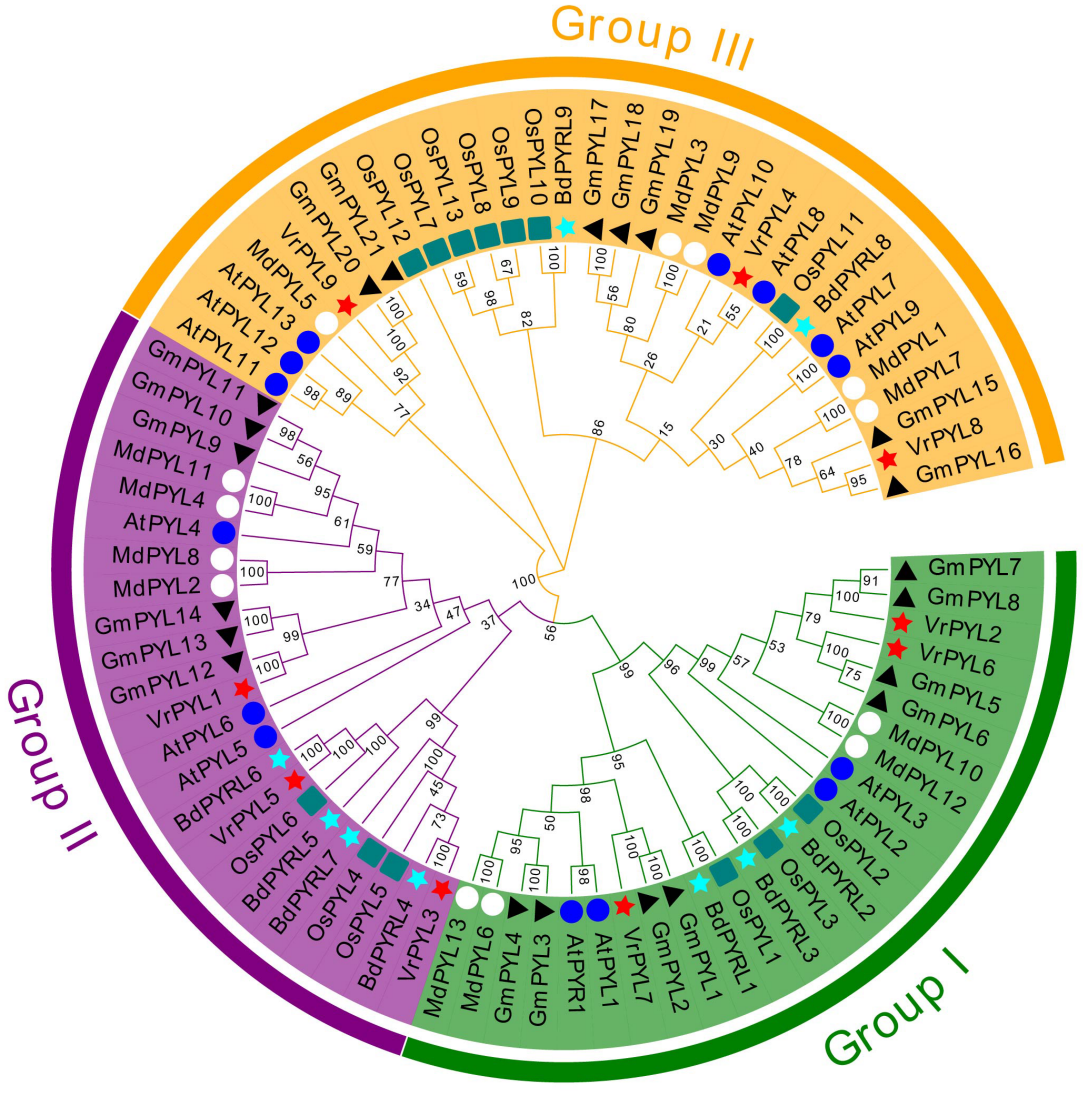
Phylogenetic tree analysis of PYL proteins of mung bean, *Arabidopsis thaliana*, rice, soybean, *Brachypodium distachyon* and apple. The tree is clustered into three distinct clades (designated as Group I, Group II, and Group III), which are colored green, purple, and yellow, respectively. PYL proteins from different species are denoted by species-specific colored symbols: VrPYL proteins by red stars, AtPYL by blue circles, OsPYL by teal rectangles, GmPYL by black triangles, BdPYL by cyan stars, and MdPYL by white circles.

For this analysis, an unrooted phylogenetic tree was built with nine VrPYL sequences from mung bean, 14 AtPYL from *A. thaliana*, 13 OsPYL from rice, 21 GmPYL from soybean, 9 BdPYL from *B. distachyon*, and 13 MdPYL from apple. The results showed that the 79 PYL sequences from these six plant species were clustered into three distinct groups (Fig. 1): Group I (25 members), Group II (23 members), and Group III (31 members), respectively. Each group comprised three VrPYL members. Notably, all groups contained PYL from both dicotyledonous and monocotyledonous plants, indicating that these PYL proteins existed prior to the divergence of dicots and monocots.

### Conserved sequences and CL2 region/loop analysis of VrPYL proteins

**Figure 2:**
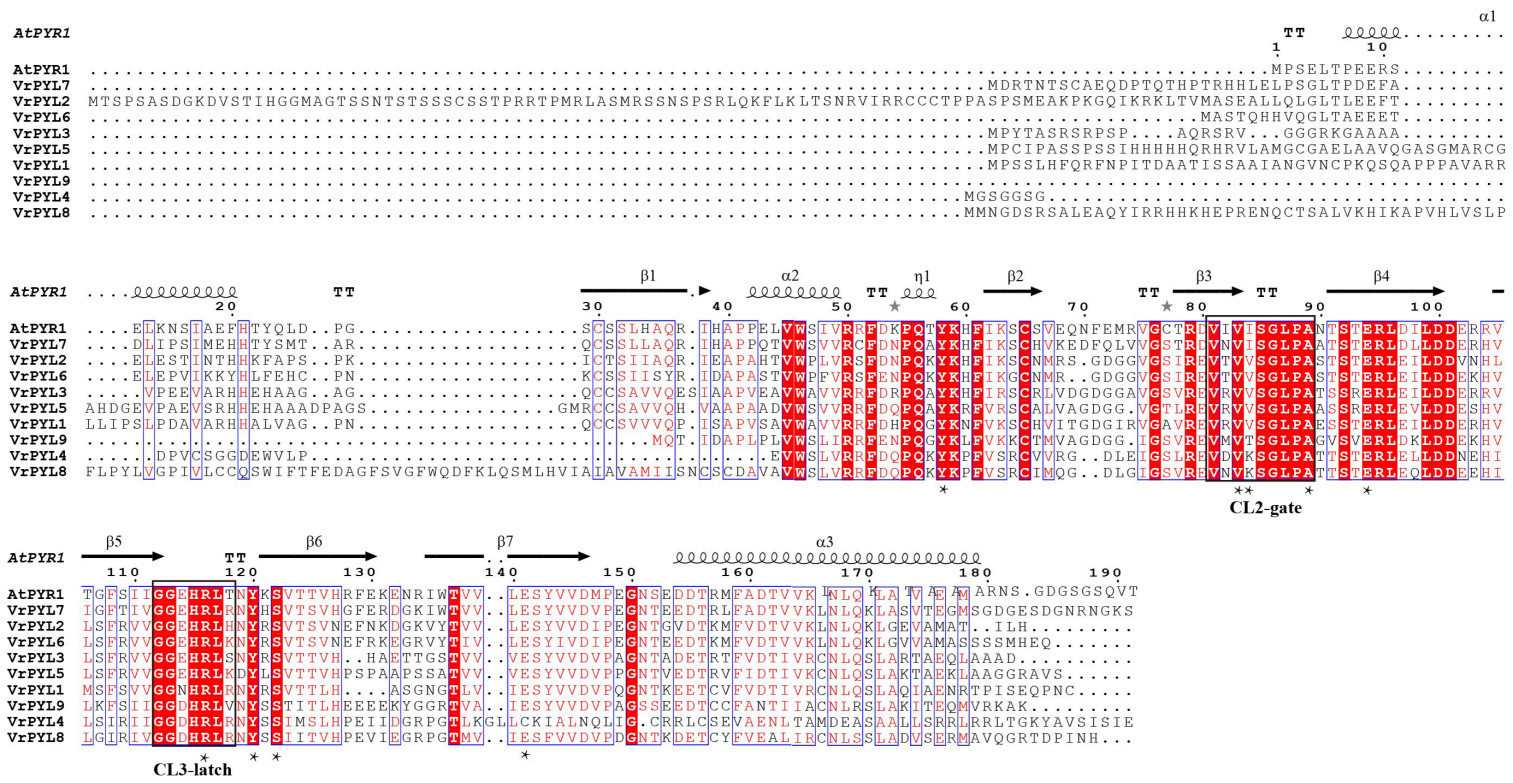
Multiple sequence alignment and secondary structure prediction of VrPYL proteins. Secondary structural elements are annotated above the primary sequence: black helices denote α-helices, black arrows denote β-sheet, and TT denotes β-turn. Black boxes mark the CL2-gate and CL3-latch. Black asterisks denote conserved amino acid residues involved in ABA recognition and binding.

Amino acid sequence alignment demonstrated that all identified VrPYL proteins harbored the signature “helix-clamp” fold of the *PYL* gene family. The core architecture of this fold comprises a structural framework of three α-helices alternating with seven β-strands, interconnected by conserved CL regions/loops. These α-helices, β-strands, and CL regions fold cooperatively to form a unique clamp-like spatial conformation that functions as the specific binding pocket for ABA (Fig. 2). Structural biology and functional validation analyses have established that the third and fourth amino acid positions in the CL2 region of PYL proteins are pivotal for regulating conformational stability, ABA-dependent signal transduction, and protein activity (Hao et al., 2011; Santiago et al., 2012). Sequence analysis of the conserved CL2 domain of VrPYL proteins demonstrated that the residue combination at positions 3 and 4 was predominantly valine-valine (VV), whereas alternative combinations were valine-lysine (VK, VrPYL4/6), valine-isoleucine (VI, VrPYL7) and valine-threonine (VT, VrPYL9). Beyond the CL2 domain, additional key amino acid residues involved in ABA interaction demonstrated high conservation among VrPYL proteins. These residues include K59, A89, E94, R116, Y120, S122 and E141 (Fig. 2).

**Table 1:** Genomic information and protein characteristics of *VrPYL* gene family.

**Gene name**	**Gene ID**	**Chromosome:location**	**CDS (bp)**	**AA**	**Molecular weight** **(kDa)**	**pI**	**Subcellular localization**	**Grand average of hydropathicity**
*VrPYL1*	Vradi02g13150	2:23335253-23335915	663	220	23.75	8.22	Cytoplasm	−0.058
*VrPYL2*	Vradi05g11800	5:20720528-20722115	843	280	30.58	9.35	Chloroplast	−0.279
*VrPYL3*	Vradi06g04160	6:4848282-4849680	594	197	21.26	7.11	Cytoplasm	−0.269
*VrPYL4*	Vradi06g10200	6:22419005-22421988	507	168	18.5	6.83	Nucleus	−0.152
*VrPYL5*	Vradi06g11870	6:28652048-28661375	834	277	31.58	4.72	Cytoplasm	−0.050
*VrPYL6*	Vradi06g14140	6:33708044-33708613	570	189	21.29	6.07	Chloroplast	−0.370
*VrPYL7*	Vradi07g07630	7:17881455-17882105	651	216	24.08	5.47	Cytoplasm	−0.487
*VrPYL8*	Vradi09g03420	9:43802800-4283777	747	248	27.8	6.05	Chloroplast	−0.032
*VrPYL9*	Vradi10g10110	10:17726678-17727124	447	148	16.29	5.93	Cytoplasm	−0.098

### Synteny analysis of the *VrPYL* gene family

**Figure 3:**
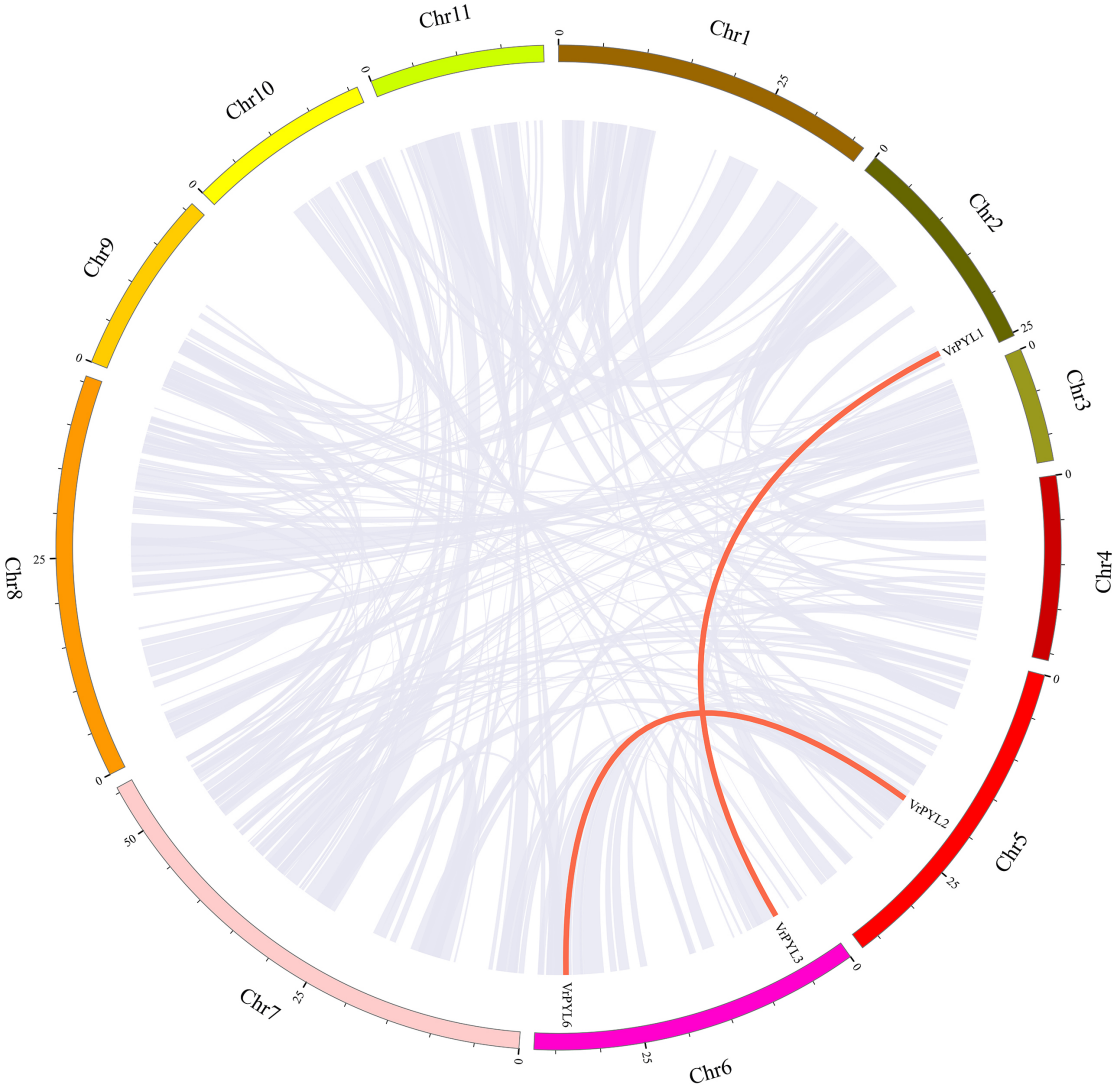
Genome-wide syntenic analysis of the *VrPYL* gene family. Outer circular track represents 11 *Vigna radiata* chromosomes, each differentiated by a unique color; numerical markers along each chromosome indicate relative physical position. Gray lines represent syntenic homologous gene pairs distributed genome-wide between different chromosomes. Highlighted red lines specifically denote syntenic relationships among *VrPYL* genes.

Gene families can expand during evolution *via* tandem and segmental duplication (Zhang, 2003). Chromosomal localization analysis revealed no tandem duplication pairs within the *VrPYL* gene family (Data S3). Segmental duplication event analysis performed with MCScanX and Circos software identified two pairs of syntenic *VrPYL* genes in mung bean (Fig. 3): *VrPYL1*/*VrPYL3* and *VrPYL2*/*VrPYL6*, both of which were derived from interchromosomal segmental duplication. The Ka/Ks ratio of intragenomic syntenic gene pairs was analyzed to investigate the characteristics of divergence and selection pressure associated with *VrPYL* gene duplication (Table 2). All Ka/Ks values were less than 1, suggesting that these *VrPYL* genes have mainly undergone purifying selection during evolution. This may have helped maintain the core functions of the *PYL* gene family.

**Figure 4:**
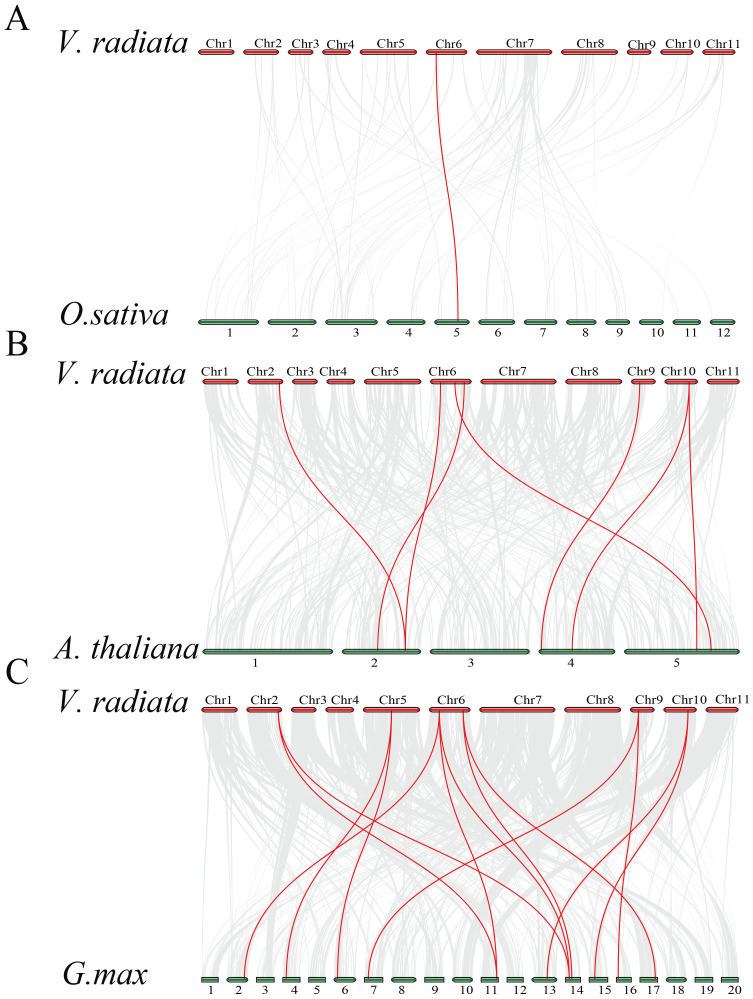
Interspecific syntenic analysis of the *VrPYL* gene family with three plant species. A, B, and C respectively depict syntenic relationships between *Vigna radiata* and *Oryza sativa*, *Arabidopsis thaliana*, and *Glycine max*. Gray lines represent genome-wide syntenic homologous gene pairs between species, while red lines specifically denote syntenic pairs of *VrPYL* genes and their orthologs in the corresponding species.

To clarify the evolutionary mechanism underlying the *VrPYL* gene family, we generated comparative synteny maps between mung bean and three representative species: the monocot rice, the dicot *A. thaliana*, and the legume soybean. Comparative analysis revealed one, seven, and thirteen syntenic gene pairs between mung bean and rice, *A. thaliana*, and soybean, respectively. Specifically, *VrPYL3* harbored syntenic homologs in all three tested species; *VrPYL1*, *VrPYL6*, *VrPYL8*, and *VrPYL9* shared syntenic homologs with *A. thaliana* and soybean; *VrPYL4* only exhibited a syntenic pair with *A. thaliana*; *VrPYL2* had a syntenic counterpart exclusively in soybean; and no syntenic homologs were detected for *VrPYL5* or *VrPYL7* among the three species (Fig. 4, Data S4). These findings indicate that most members of the *VrPYL* gene family have been relatively conserved throughout the evolutionary process; this may have contributed to the adaptive responses of mung bean to diverse environmental stimuli.

### Gene structure and conserved motif analysis of the *VrPYL* gene family

**Figure 5:**
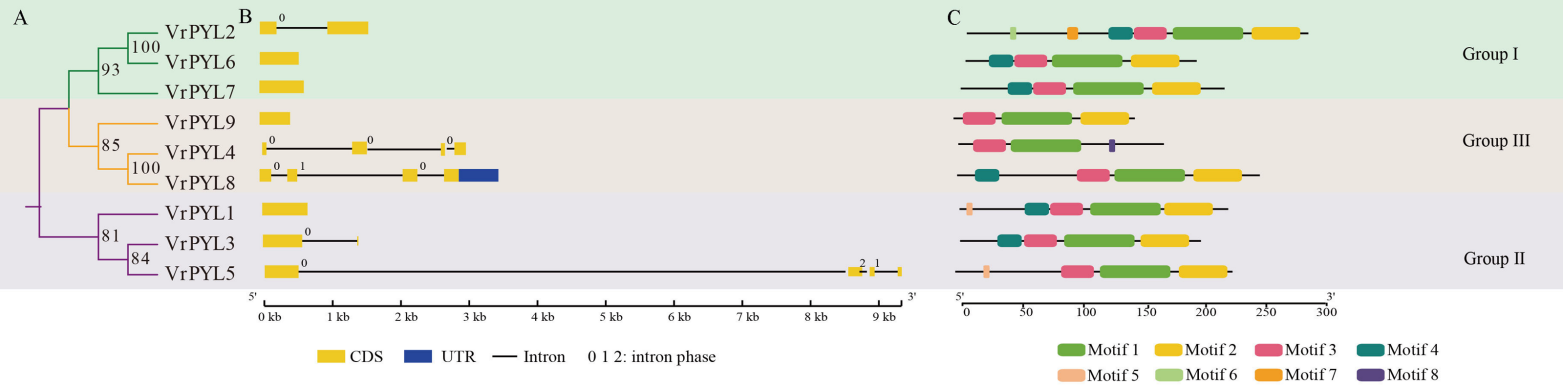
Phylogenetic tree, gene structure, and conserved motif analyses of the *VrPYL* gene family. (A) Phylogenetic tree of VrPYL constructed using the maximum likelihood method. (B) Exon-intron architectures of *VrPYL* genes. Yellow boxes indicate protein-coding domains (CDSs), solid lines represent introns, and light blue boxes indicate untranslated regions (UTRs). (C) Distributions of conserved motifs in VrPYL proteins, with each color corresponding to a unique motif.

Comprehensive analyses were performed on the phylogenetic relationships, gene structures, and conserved motifs of *VrPYL* gene family (Fig. 5). A total of nine *VrPYL* genes were phylogenetically clustered into three distinct groups, a pattern congruent with the phylogenetic classification patterns observed for *PYL* genes from other plant species (Fig. 5A). These *VrPYL* genes could be further subdivided into intron-less and intron-containing groups, with *VrPYL8* being the sole member harboring an untranslated region (Fig. 5B). Within Group I, all *VrPYL* genes contained 1–2 exons without flanking regulatory sequences. Only *VrPYL2* possessed an intronic region. In Group II, all *VrPYL* genes lacked flanking sequences, and *VrPYL3* and *VrPYL5* contained introns. For Group III, *VrPYL4* and *VrPYL8* exhibited a gene structure consisting of four exons and three introns, whereas *VrPYL9* was classified as intron-less. Notably, *VrPYL5* was the longest among all *VrPYL* genes, exceeding 9,000 bp (Fig. 5B). Within each group, the *VrPYL* genes shared conserved exon-intron organization patterns, indicating close evolutionary affinities among these genes and validating the rationality of the group classification scheme.

Conserved motif analysis of VrPYL protein sequences was conducted using the MEME Suite web server. The results identified eight distinct conserved motifs (Fig. 5C, Data S5). All VrPYL protein sequences contained Motifs 1–3, with the exception of VrPYL4, which lacked Motif 2. This observation reflects a high degree of conservation in the amino acid sequences of VrPYL proteins. In addition to the shared conserved motifs, each group possessed unique conserved motifs: VrPYL2 in Group I contained Motifs 6 and 7; VrPYL1 and VrPYL5 in Group II harbored Motif 5, and VrPYL4 in Group III contained Motif 8. Collectively, the conservation of motif profiles among proteins within the same group suggests potential functional redundancy or similarity, while the presence of group-specific unique motifs implies that these proteins may exert distinct biological functions in response to specific physiological or environmental cues.

### Analysis of the cis-acting element in the *VrPYL* promoter

**Figure 6:**
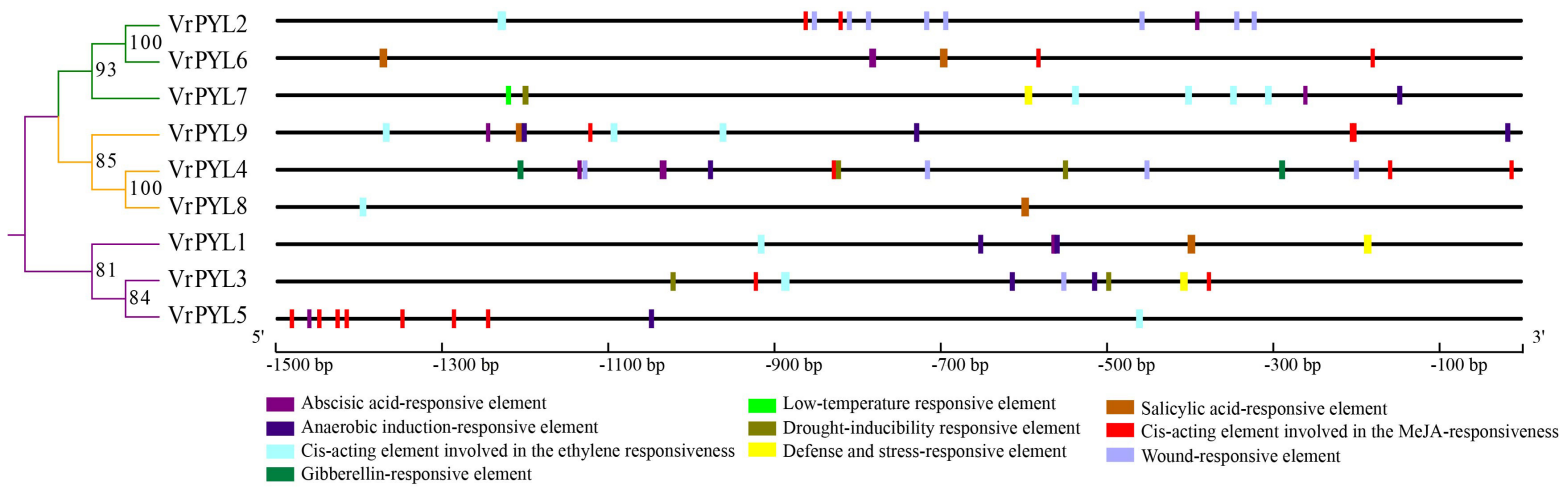
Distribution of cis-acting elements in promoters of the *VrPYL* genes. Different colored squares indicate distinct types of cis-acting elements.

To investigate the responsiveness of *VrPYL* genes to environmental signals, a bioinformatic analysis was performed on cis-acting elements within the 1,500 bp upstream sequence of the start codon (ATG) of *VrPYL* genes. The promoter regions of the *VrPYL* genes harbored abundant cis-acting elements (Fig. 6), among which hormone-responsive and stress-related elements were the focus in this study. Five types of stress-related elements were identified, including defense and stress responsiveness elements, drought inducibility-responsive element, low-temperature responsive element, anaerobic induction-responsive element, and wound-responsive element. Furthermore, various plant hormone-responsive elements were identified, including those responsive to abscisic acid, ethylene, gibberellin, methyl jasmonate and salicylic acid. Notably, the abscisic acid-responsive elements were ubiquitously distributed in the promoters of all *VrPYL* genes, with the exceptions of *VrPYL3* and *VrPYL8* (Fig. 6, Data S6).

**Table 2:** Ka/Ks analysis for duplicated gene pairs of *VrPYL* in mung bean.

**Duplicated gene 1**	**Duplicated gene 2**	**Type of duplication**		**Ka**	**Ks**	**Ka/Ks**	**Purifying selection**
*VrPYL1*	*VrPYL3*	Segmental	0.136537034		0.952184751	0.143393427	Yes
*VrPYL2*	*VrPYL6*	Segmental	0.168895621		1.338599714	0.126173358	Yes

### Expression responses of *VrPYL* genes to abiotic stresses and ABA treatment

**Figure 7:**
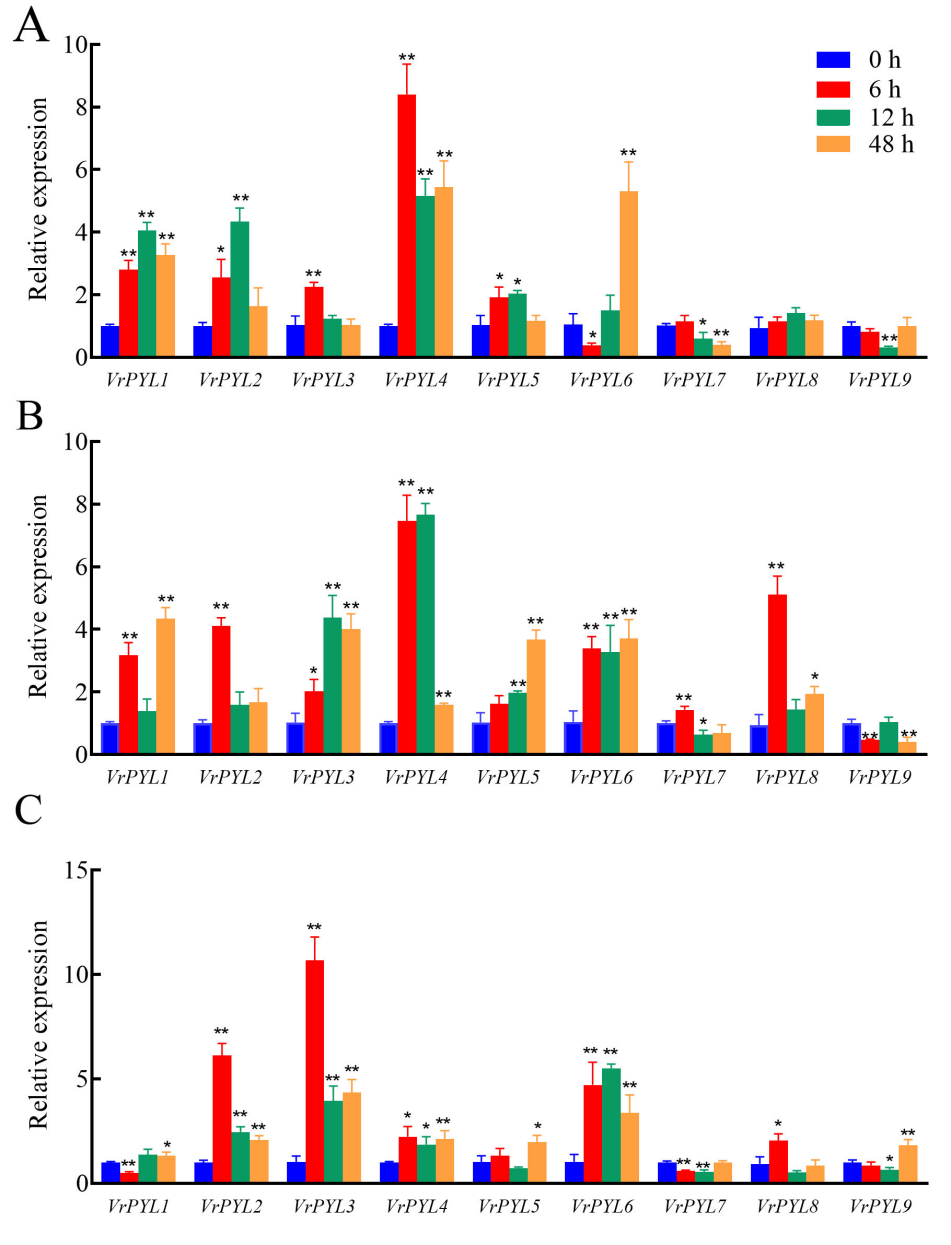
Expression profiles of *VrPYL* genes in response to various treatments. (A) 100 µmol/L ABA, (B) 15% PEG, (C) 100 mmol/L NaCl. The relative expression levels of *VrPYL* genes were determined at 6, 12, and 48 h following treatment, with expression levels normalized to those at 0 h. All values represent the mean ± standard error (SE) of three independent biological replicates. Asterisks indicate values that are significantly different from CK (0 h) (* *p* < 0.05, ** *p* < 0.01, one-way ANOVA).

As ABA receptors, PYL proteins serve crucial functions in plant signaling pathways that mediate responses to abiotic stress. To elucidate the expression profiles of *VrPYL* genes in response to diverse abiotic stresses, qRT-PCR was employed to determine the transcriptional levels of nine *VrPYL* genes. The results (Fig. 7A) demonstrated that under 100 µmol/L ABA treatment, *VrPYL1* and *VrPYL4* exhibited upregulated expression at 6 h, 12 h, and 48 h following treatment, with *VrPYL4* displaying the most prominent upregulation (8-fold higher at 6 h than the control). *VrPYL2*, *VrPYL3*, and *VrPYL5* were upregulated at 6 h but reverted to basal levels (consistent with the control) by 48 h. *VrPYL7* underwent significant downregulation at 12 h and 48 h, while *VrPYL8* maintained relatively stable transcriptional levels throughout the treatment period.

Under 15% PEG treatment, most of the *VrPYL* genes were upregulated, with the exception of *VrPYL9*. *VrPYL4* showed the highest transcriptional activity, reaching approximately 8-fold higher than that of the control at 6 h and 12 h following treatment (Fig. 7B). Under 100 mmol/L NaCl treatment, *VrPYL2*, *VrPYL3*, *VrPYL4*, and *VrPYL6* displayed sustained upregulation at all three tested time points. *VrPYL3* exhibited the highest upregulation, with a transcriptional level approximately 10-fold higher than the control at 6 h. *VrPYL7* was significantly downregulated at 6 h and 12 h following treatment (Fig. 7C). Collectively, these findings suggest that most *VrPYL* genes in mung bean mediate responses to abiotic stresses and ABA-dependent signal transduction pathways.

## Discussion

ABA is a central phytohormone responsible for regulating plant growth and developmental processes, and it also mediates adaptive responses to various environmental adversities. The activation of the ABA signaling pathway is dependent on the PYL receptor family (Kavi Kishor et al., 2022). The *PYL* gene family has been identified in multiple plant species, whereas the *PYL* gene family in mung bean remained uncharacterized and understudied. Herein, genome-wide screening identified nine *PYL* genes (*VrPYL1*–*VrPYL9*) in mung bean. This number is lower than in *A. thaliana* (14), *O. sativa* (13), and *G. max* (21); the differences may be associated with genome duplication events during species evolution and adaptation to diverse environmental conditions. Furthermore, our results demonstrated that the nine *VrPYL* genes were unevenly distributed across six of the 11 mung bean chromosomes, with no homologous copies detected on the remaining five chromosomes. The widespread absence of *PYL* genes on nearly half of the chromosomes could be mainly attributed to whole-genome duplication (WGD) events, together with subsequent fractionation and chromosomal rearrangements during the evolutionary divergence of mung bean from its closely related legume species, which generally drive the differential retention and loss of duplicated genes at distinct chromosomal locations (Cannon et al., 2006; Freeling, 2009). Notably, most *VrPYL* genes were located in the terminal regions of chromosomes, which are well-recognized recombination hotspots in plant genomes (Henderson & Bomblies, 2021). This positional preference may confer evolutionary adaptability to the *PYL* gene family, since high recombination frequencies facilitate the generation of genetic diversity and accelerate adaptive evolution in response to diverse abiotic stresses (Chen et al., 2018; Henderson & Bomblies, 2021).

A physicochemical property analysis (Table 1) demonstrated significant variation in the lengths of VrPYL proteins (148–280 aa), MW (16.29–31.58 kDa) and pI (4.72–9.35). Notably, VrPYL1, VrPYL2, and VrPYL3 are predicted to be alkaline proteins (pI > 7), whereas the majority of other VrPYL members exhibit acidic properties. Although the core interaction between PYL receptors and PP2Cs relies on abscisic acid (ABA)-induced conformational changes, the unique surface charge characteristics endowed by the alkaline isoelectric point may finely modulate their binding affinity (Grassmann et al., 2023). Furthermore, the alkaline physicochemical properties of VrPYL1, VrPYL2, and VrPYL3 not only facilitate their adaptation to the pH microenvironments of specific subcellular compartments, but also help maintain protein solubility and structural integrity when intracellular pH homeostasis is disrupted by abiotic stresses (Panja, Maiti & Bandyopadhyay, 2020). Most VrPYL proteins are hydrophilic (grand average of hydropathicity < 0), consistent with the hydrophilic characteristics of PYL proteins in *A. thaliana* (Ma et al., 2009; Park et al., 2009) and *Helianthus annuus* (Wang et al., 2024). This suggests that hydrophilicity may serve as a structural basis for PYL proteins to function as ABA receptors and participate in signal transduction, as their hydrophilic nature facilitates interactions with other signaling molecules in aqueous environments such as the cytoplasm and chloroplasts. Additionally, bioinformatic prediction using the PLoc server indicated that most VrPYL proteins were predicted to localize in the chloroplasts and cytoplasm, whereas only VrPYL4 was predicted to be localized in the nucleus. Such differences in localization may be linked to functional specialization; this aligns with previous reports that PYL proteins are predominantly localized in the cytoplasm and chloroplasts in *Cucumis sativus* (Zhang et al., 2022) and *H. annuus* (Wang et al., 2024). The subcellular localization results in this study were solely derived from bioinformatic predictions using the PLoc server. The predicted nuclear localization of VrPYL4 constitutes a distinct characteristic that sets it apart from other family members, implying that it may exert specialized biological functions in nuclear ABA signaling pathways. Although previous studies in *Arabidopsis* have identified nuclear-localized PYL proteins that participate in the transcriptional regulation of stress-responsive genes (Park et al., 2009), further experimental evidence is still required to verify the subcellular localization of VrPYL4 and elucidate its functional roles in mung bean.

Phylogenetic analysis classified 79 PYL members from six plant species into three groups. Each group contained PYL from both dicotyledonous and monocotyledonous plants, indicating that the divergence of the three PYL groups occurred prior to the split between monocots and dicots. Furthermore, VrPYL members showed a closer phylogenetic relationship to GmPYL, further verifying the evolutionary relatedness of PYL among leguminous plants. For example, VrPYL3 shared high homology with GmPYL8. Previous studies have shown that GmPYL8, an upstream receptor in the ABA signaling pathway, can promote stomatal closure in soybean, thereby reducing water loss and enhancing drought tolerance in transgenic soybean lines (Wei et al., 2019). In the present study, *VrPYL3* responded to drought stress, implying that it may exert similar functions. The functional conservation of homologous genes across species provides a basis for exploring stress-resistant genes in mung bean through homologous gene analysis.

Key factors contributing to gene family expansion include segmental duplication, tandem duplication, and transposition events (Kong et al., 2007). Analysis of the expansion mechanism revealed no tandem duplication in the *VrPYL* gene family, but two pairs of intraspecific syntenic genes (*VrPYL1*/*VrPYL3* and *VrPYL2*/*VrPYL6*) (Fig. 3), both appearing to be derived from inter-chromosomal segmental duplication events. This suggests that segmental duplication has served as the key driver behind the expansion of the *VrPYL* gene family, consistent with previous findings in *H. annuus* (Wang et al., 2024) and *Gossypium hirsutum* (Chen et al., 2017). Further selection pressure analysis showed that the Ka/Ks values of all gene pairs were less than 1 (Table 2), indicating that *VrPYL* genes have been under strong purifying selection during evolution. This selection pressure effectively maintains the core functions of the genes, consistent with the purifying selection characteristics of *PYL* genes in *A. thaliana* (Park et al., 2009) and *O. sativa* (Yadav et al., 2020) and reflecting the evolutionary conservation of the *PYL* gene family. Interspecific synteny analysis (Fig. 4) demonstrated that the number of syntenic gene pairs between mung bean and *G. max* (13 pairs) was significantly higher than that between mung bean and *A. thaliana* (seven pairs) or *O. sativa* (one pair), indicating a closer evolutionary relationship between mung bean and *G. max*. Notably, genes homologous to *VrPYL3* were present in all three species, suggesting that *VrPYL3* existed prior to species divergence and is highly conserved. In contrast, no syntenic genes of *VrPYL5*/*7* were identified in other species, suggesting that they may be novel genes generated during mung bean evolution and that they may provide unique functions for mung bean to adapt to specific environments.

The *PYL* gene family serves as core receptors in the plant ABA signaling pathway, and the CL2 loop within PYL proteins is a key functional region (Park et al., 2009). Localized in the ligand-binding pocket, the CL2 loop specifically binds ABA through hydrophobic interactions and hydrogen bonds, directly determining binding affinity. Additionally, the CL2 loop can drive the conformational rearrangement of PYL from inactive dimers to active monomers that further interact with downstream PP2C phosphatases, thereby relieving the inhibitory effect of PP2Cs on ABA signaling and initiating stress responses (Yin et al., 2009). Consistent with the conserved residue combinations at positions 3 and 4 of the CL2 loop reported in *A. thaliana*, *G. max* and tobacco (Bai et al., 2019; Bai et al., 2013; Santiago et al., 2012), the CL2 domain of VrPYL proteins also displays evolutionary conservation. Notably, VrPYL proteins predominantly harbor the VV combination at these two positions, with specific variants including VK (VrPYL4/6), VI (VrPYL7) and VT (VrPYL9). Among these, the VT combination represents a mung bean-specific polymorphic site that has not been documented in other plant species, suggesting that VrPYL proteins may modulate their interactions with ABA and PP2Cs through residue variations in the CL2 loop while retaining fundamental ABA-binding activity, thereby conferring more flexible signal regulatory functions.

The gene structure analysis showed that the *VrPYL* genes could be divided into two groups: the intron-less group (*VrPYL1*, *VrPYL6*, *VrPYL7*, and *VrPYL9*) and the intron-containing group (*VrPYL2*, *VrPYL3*, *VrPYL4*, *VrPYL5*, and *VrPYL8*) (Fig. 5). Members within the same group exhibited analogous exon-intron structures. For example, *VrPYL4*/*8* in Group III both contain four exons and three introns, reflecting the co-evolutionary relationship between gene structure and group classification. The presence of introns may enhance gene functional diversity through post-transcriptional regulation (*e.g.*, alternative splicing) (Gallegos & Rose, 2019), suggesting that intron-containing *VrPYL* genes may be involved in multiple biological processes. Notably, the genomic length of *VrPYL5* (>9 kb) is markedly longer than that of other *VrPYL* family members (Fig. 5B). This size expansion is not attributed to alterations in the lengths of coding sequences (CDS) or untranslated regions (UTR), but is primarily driven by extensive intron expansion, likely mediated by the insertion of transposable elements (TEs), a well-characterized mechanism underlying genome size variation in plants (Lee & Kim, 2014). Gene duplication events may further contribute to structural divergence by introducing additional non-coding sequences into intronic regions (Xu et al., 2012). Conserved motif analysis identified eight motifs, among which motifs 1–3 were core motifs of VrPYL proteins (except VrPYL4 lacked motif 2). These core motifs are speculated to form the “helix-clamp” domain of PYL proteins, providing key structural support for ABA binding and PP2Cs interaction. Motifs 6/7, motif 5, and motif 8 are exclusive to Group I, Group II, and Group III, respectively. These unique motifs may confer distinct functions to VrPYL proteins.

Cis-acting element analysis of *VrPYL* gene promoters (Fig. 6) revealed a wealth of hormone-responsive and abiotic stress-responsive elements. These cis-acting elements were consistent with those identified in other species, indicating that *PYL* genes across different plants share analogous transcriptional response profiles (Yadav et al., 2020; Zhou et al., 2023). All *VrPYL* genes contained ABA-responsive elements (ABREs) except *VrPYL3*/*8*, consistent with the qRT-PCR results showing that most *VrPYL* genes responded to ABA treatment. Previous studies have indicated that the absence of ABREs in the promoter is often associated with an unresponsive expression pattern to ABA (Fujita et al., 2009). In this study, *VrPYL8* lacked ABREs in its promoter and exhibited no significant change in expression under ABA treatment, and this correlation suggests that ABREs may serve as important cis-elements for the ABA-induced expression of *VrPYL* genes. Notably, although *VrPYL3* lacked ABREs in its promoter, it exhibited marked transcriptional upregulation at 6 h following ABA treatment (Fig. 7A), implying that this gene responds to ABA signaling through an ABRE-independent regulatory pathway, a mechanism documented in several *PYL* family members (Li et al., 2013). Accordingly, we hypothesize that *VrPYL8* participates in constitutive ABA signaling, or mediates cross-regulation between the ABA signaling cascade and other signaling pathways, whereas *VrPYL3* presumably mediates ABRE-independent ABA-induced transcription *via* alternative stress-responsive cis-elements or transcription factors within its promoter.

ABA is a key hormone that helps plants cope with abiotic stress. When plants are subjected to stressful environments, endogenous ABA levels rapidly accumulate, activating the ABA signaling pathway and further inducing resistance mechanisms (Sah, Reddy & Li, 2016). The qRT-PCR results showed that most *VrPYL* genes exhibited significant expression responses to ABA, PEG-6000, and NaCl stress, with distinct response patterns across genes, reflecting functional differentiation of the *VrPYL* gene family in abiotic stress responses. Under ABA treatment, *VrPYL1*/*4* were continuously upregulated, while the expression levels of *VrPYL2*/*3*/*5* were upregulated in the short term and then declined (Fig. 7). This indicates that *VrPYL1*/*4* may act as core responsive genes in the ABA signaling pathway, whereas *VrPYL2*/*3*/*5* may be involved in early ABA signal transduction to enhance mung bean adaptability to stress. Under PEG treatment, most *VrPYL* genes were upregulated except *VrPYL9*, with *VrPYL4* showing the highest expression level (approximately 8-fold higher than the control at 6 h and 12 h). This transient transcriptional pattern is characteristic of early stress-responsive genes: rapid upregulation likely serves to quickly activate ABA-dependent drought tolerance pathways, while subsequent downregulation may reflect stress adaptation or negative feedback mechanisms. Given the presence of drought-inducible responsive elements in its promoter, *VrPYL4* is speculated to be a key gene in mung bean drought tolerance responses. Under NaCl treatment, *VrPYL2*/*3*/*4*/*6* were upregulated across all tested time points, with *VrPYL3* displaying a prominent transient peak at 6 h (approximately 10-fold higher than the control), followed by reduced expression at 12 h and 48 h. This early peak suggests that *VrPYL3* may act as a core early trigger for salt stress signaling, initiating downstream adaptive responses to mitigate ionic stress. VrPYL7 was downregulated under both ABA and NaCl treatments, which may be related to negative regulation in response to stress. Additionally, most *VrPYL* genes responded to multiple stresses, indicating that they may be involved in cross-stress resistance regulation and enhance mung bean adaptability to complex environments through shared signaling pathways. Although this early peak-then-decline expression pattern is a typical feature of plant stress signaling regulators, the present study was only focused on transcriptional-level analysis and lacks relevant evidence at the protein level as well as physiological determinations. Therefore, future studies will measure key physiological stress indices in transgenic or gene-silenced lines to verify whether these early transcriptional responses contribute to enhanced stress tolerance in plants.

Taken together, this study provides genome-wide identification and systematic characterization of the *PYL* gene family in mung bean, an important food legume crop. We clarified the molecular characteristics and stress-responsive expression patterns of this family, and identified *VrPYL3* and *VrPYL4* as candidate genes for stress resistance. These findings offer valuable genetic resources and a theoretical basis for future functional research and molecular breeding of mung bean.

## Conclusions

In this study, a total of nine *VrPYL* genes (*VrPYL1*–*VrPYL9*) were identified in mung bean (*Vigna radiata* L.) through genome-wide analysis. Their encoded proteins were predicted to be hydrophilic and predominantly localized to the chloroplasts and cytoplasm, with only VrPYL4 being predicted to localize to the nucleus. The nine *VrPYL* genes were unequally distributed on chromosomes 2, 5, 6, 7, 9, and 10, among which chromosome 6 was is enriched with four genes (*VrPYL3*/*4*/*5*/*6*). Phylogenetic analysis revealed that *PYL* genes from different plant species were clustered into three groups. Segmental duplication was the primary driver of the expansion of this gene family, with duplicated gene pairs having undergone purifying selection during evolution. Sequence feature analysis showed that all VrPYL proteins contained the family-specific “helix-clamp” domain and highly conserved key ABA-binding residues, and their gene structures and conserved motifs exhibit distinct group-specific characteristics. Promoter element prediction indicated that the promoter regions of the *VrPYL* genes contained abundant hormone-responsive and stress-related elements. Furthermore, qRT-PCR analysis revealed that *VrPYL* genes (especially *VrPYL3*/*4*) exhibited significant transcriptional responses to abiotic stresses and ABA treatment, suggesting that these genes represent promising candidate genes for stress resistance research whose functions require further experimental validation. This study provides a foundation for in-depth investigation of the functions of *PYL* genes in mung bean.

## Supplemental Information

10.7717/peerj.21432/supp-1 Supplemental Information 1 PYL protein sequences from different species

10.7717/peerj.21432/supp-2 Supplemental Information 2 PCR primers used for qRT-PCR in this study

10.7717/peerj.21432/supp-3 Supplemental Information 3 Distribution of *VrPYL* genes on mung bean chromosomes

10.7717/peerj.21432/supp-4 Supplemental Information 4 Synteny analysis of *PYL* genes between mung bean and other plant species

10.7717/peerj.21432/supp-5 Supplemental Information 5 The sequences and logos of the 8 motifs

10.7717/peerj.21432/supp-6 Supplemental Information 6 Positions of cis-acting elements present in the 1.5 kb upstream promoter of the *VrPYL* genes

10.7717/peerj.21432/supp-7 Supplemental Information 7 Raw data of qRT-PCR

10.7717/peerj.21432/supp-8 Supplemental Information 8 MIQE checklist

10.7717/peerj.21432/supp-9 Supplemental Information 9 Code Scripts and Docker Desktop run commands.

## Additional Information and Declarations

### Data Availability

The following information was supplied regarding data availability: The code and raw measurements are available in the Supplemental Files.
