# Supplementary material for: Characterization, evolutionary analysis, and expression profiling of the VrPYL gene family in mung bean in response to abiotic stress
Source: PeerJ. 2026 Jun 22;14:e21432. doi: 10.7717/peerj.21432 (PMC13296803; doi:10.7717/peerj.21432)
Supplement: Supplemental Information 2 [file peerj-14-21432-s002.docx]

**Supplemental Data S2: PCR primers used for qRT-PCR in this study.**

| **Gene name** | **Forward（5′-3′）** | **Reverse（5′-3′）** |
| --- | --- | --- |
| *VrPYL1* | TATCCATCGACCCTGCTCGA | GCCACTCAACTTCACACCCT |
| *VrPYL2* | GGAACCACCGGCTGAGAAAT | ACACGCACGTTTCCTCCTTA |
| *VrPYL3* | TTCACCGAACTGGAGAGCAC | GTACTTCTGCGGGTTGTCGA |
| *VrPYL4* | CTGCTCCGTCGTCATTCAGG | TGCTTGTACCCTTGCGGATT |
| *VrPYL5* | ACTCCTCGATGACAATGAGCA | GCAAGCCCTTCAGTGTACCT |
| *VrPYL6* | GGAAGGCAAGGTATCAGCGA | CCGTGAAGTCCGTAGCAAGT |
| *VrPYL7* | TGCAACATGAGAGGTGACGG | CCACCAACCACCCTAAAGCT |
| *VrPYL8* | TTTGTGGTGGATGTGCCTGA | GTACTTCTGCGGGTTGTCGA |
| *VrPYL9* | GCAAACAATCGACGCACCTT | TTCCAATGCCACCATCTCCG |
