# Supplementary figures and images for: Characterization, evolutionary analysis, and expression profiling of the VrPYL gene family in mung bean in response to abiotic stress
Source: PeerJ. 2026 Jun 22;14:e21432. doi: 10.7717/peerj.21432 (PMC13296803; doi:10.7717/peerj.21432)

chr2

chr5

chr6

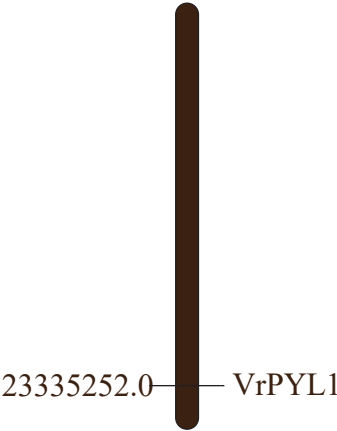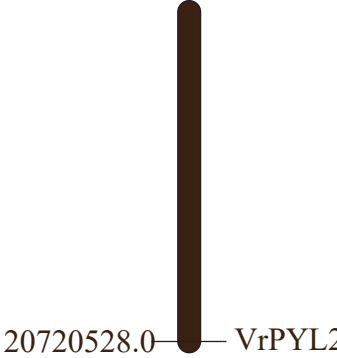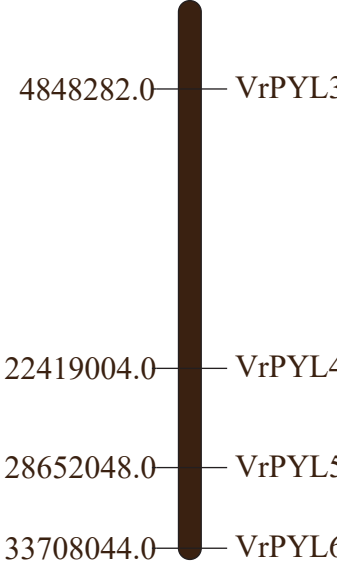

chr7

chr9

chr10

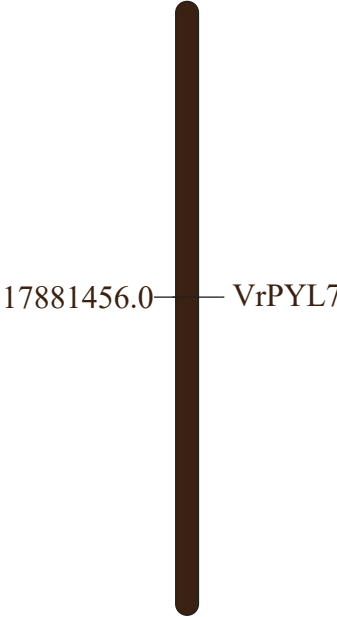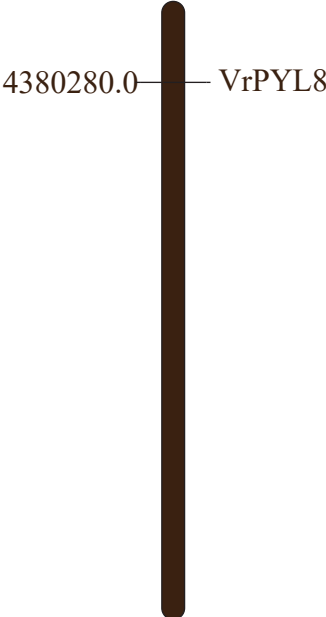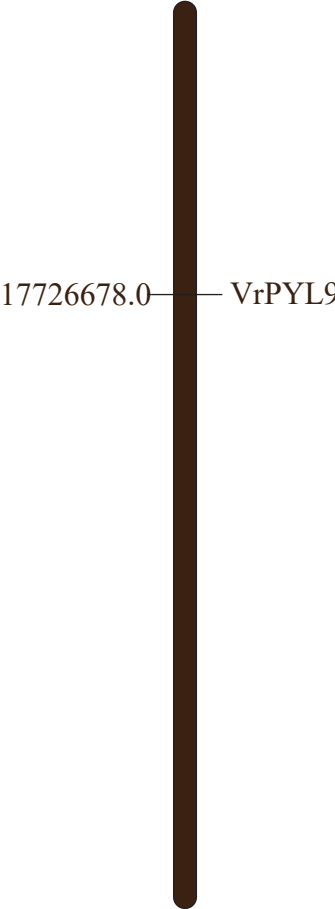

Supplement: Supplemental Information 3 [file peerj-14-21432-s003.pdf]
