# Supplementary material for: Characterization, evolutionary analysis, and expression profiling of the VrPYL gene family in mung bean in response to abiotic stress
Source: PeerJ. 2026 Jun 22;14:e21432. doi: 10.7717/peerj.21432 (PMC13296803; doi:10.7717/peerj.21432)

**Supplemental Data S5: The sequences and logos of the 8 motifs.**

| Motif | | Motif sequences |
| --- | --- | --- |
| Motif 1 | GDGGVGSVREVRVVSGLPATTSTERLEILDDERHVLSFRVVGGEHRLRNYRSVTTVHPE | |
| Motif 2 | YTVVIESYVVDVPEGNTEEDTRVFVDTIVKCNLQSLAKVAE | |
| Motif 3 | IEAPASAVWSLVRRFDNPQKYKHFVKSC | |
| Motif 4 | IARHHKYAPGPNQCSSIIVQR | |
| Motif 5 | WIFTFCDVGFNVGFWQD | |
| Motif 6 | WRRTPM | |
| Motif 7 | KPKGWIKRKL | |
| Motif 8 | GCRRFC | |

The logos of the 8 motifs.

Motif1


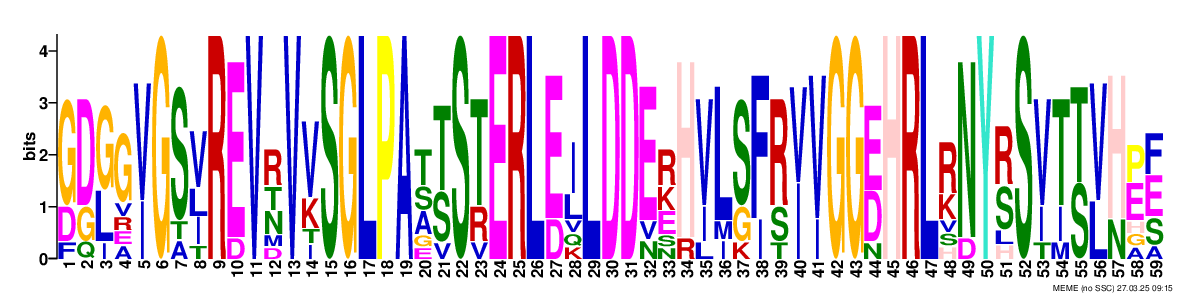


Motif2


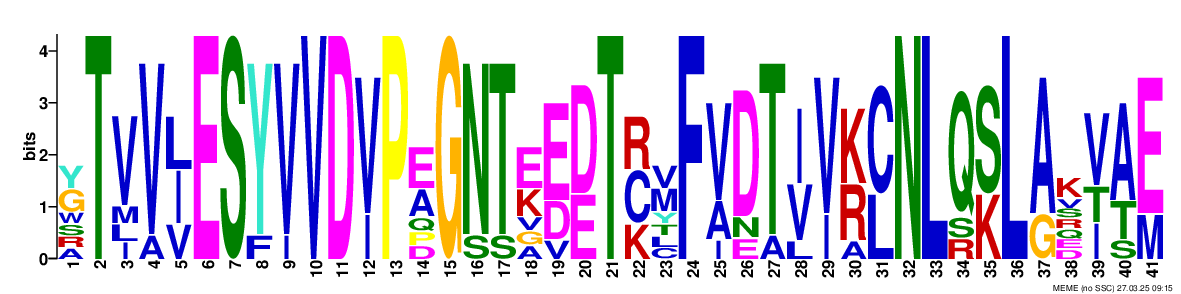


Motif3


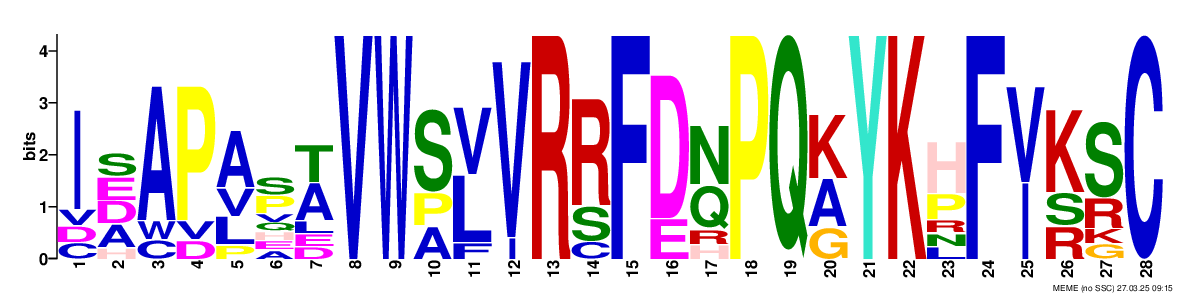


Motif4


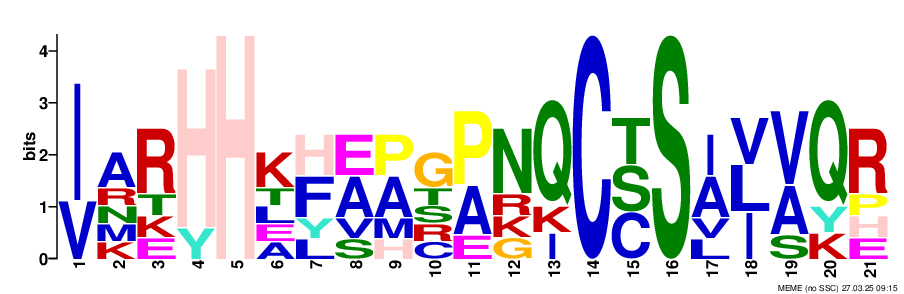


Motif5


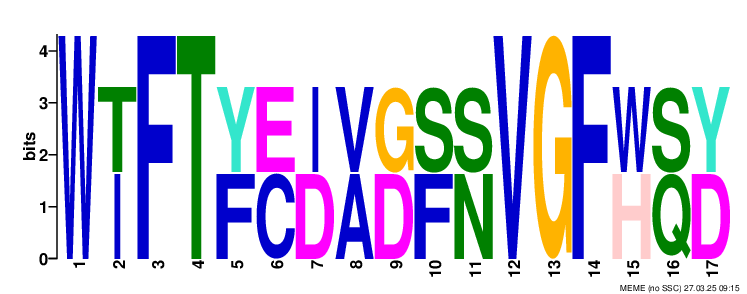


Motif6


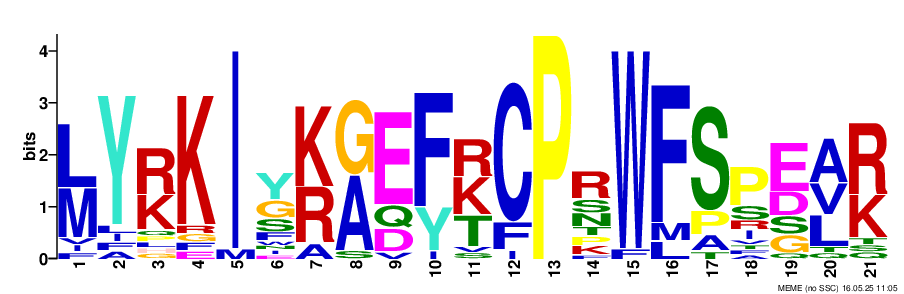
Motif7


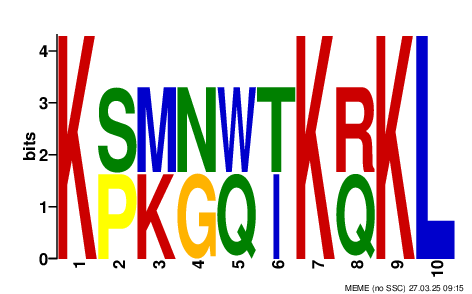


Motif8


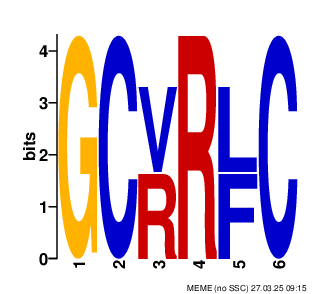

Supplement: Supplemental Information 5 [file peerj-14-21432-s005.docx]
